# Occult mortality and readmissions in a trauma intensive care unit (TICU): factors of risk

**DOI:** 10.1186/2197-425X-3-S1-A77

**Published:** 2015-10-01

**Authors:** P Ruiz de Gopegui Miguelena, A Tejada Artigas

**Affiliations:** Intensive Care Service, Hospital Universitario Miguel Servet, Zaragoza, Spain

## Introduction

Occult mortality: is the mortality which happens in the hospitalization ward after the discharge from ICU during the same hospitable admission.

Readmissions: they represent the patients who are discharged from ICU and they have to be readmitted into the ICU unexpectedly. The readmission is precocious if it occurs in the firsts 72 hours after the ICU discharge.

## Objectives

To describe and to identify those factors of risk that might associate higher risk of death and/or readmission for the patients in the moment of their ICU discharge.

## Methods

It is a retrospective study which information comes from all the patients who were admitted in the TICU from January 2011 to December 2013, and were discharged alive from TICU. They are excluded from the study all patients who were discharged from ICU with orders to limit the intensive treatment or not reanimation.

25 variables were selected to study in our patients, including the diagnosis, the number of days of orotrahceal intubation, treatment with antibiotics and/or vasopressors, blood transfusion, the presence of tracheostomy in the moment of the TICU discharge, time between extubation and TICU discharge, presence of nosocomial infection, reason of dead or readmission. The information was gathered in a database in Excel.

## Results

From January 2011 to December 2011, 1748 patients were admitted into the TICU 2013. The average of age was 56 years. The mortality in the TICU was 11.2%, so 1681 patients were discharged alive from the TICU. 23 patients (1%) represented the “occult mortality” happened during these three years. From them, 44% needed orotracheal intubation; 69% were discharged from TICU on holiday or weekend; 26% of the dead reasons were related with respiratory insufficiency, and 25% were related with infectious complications; 33% of the patients had a nosocimial infection during the TICU admission. 37 patients were readmitted in these three years, from them 19 were precocious. From the readmitted patients, 75,7% had needed orotracheal intubation; The main reasons of readmission were surgical complications (35%) and infectious complications (30%). 81% of the patients who were readmitted came from a surgical service. 40 of the patients who were readmitted precociously, were discharged from TICU on weekend. 32% of the patients who were readmitted, died in TICU.

## Conclusions

The mortality of patients who were readmitted was higher than the average of the TICU. The most important reason of occult mortality and readmission are infectious complications. The most frequent reason (60%) of readmission and/or occult mortality in patients who were in hospitalization ward more than 14 days, were infectious complications.

